# Editorial: Advances in artificial intelligence and machine learning applications for the imaging of bone and soft tissue tumors

**DOI:** 10.3389/fradi.2024.1523389

**Published:** 2024-12-17

**Authors:** Brandon K. K. Fields, Bino A. Varghese, George R. Matcuk

**Affiliations:** ^1^ Department of Radiology & Biomedical Imaging, University of California, San Francisco, San Francisco, CA, United States; ^2^ Department of Radiology, Keck School of Medicine, University of Southern California, Los Angeles, CA, United States; ^3^ Department of Imaging, Cedars-Sinai Medical Center, Los Angeles, CA, United States

**Keywords:** radiomics, texture analysis, deep learning, machine learning, artificial intelligence, cancer imaging

**Editorial on the Research Topic**
Advances in artificial intelligence and machine learning applications for the imaging of bone and soft tissue tumors

Growing interest in artificial intelligence (AI) applications in the biological and medical sciences has led to a notable surge in related publications in recent years. A PubMed query for “machine learning” yielded 33,855 results published in the year of 2023 alone, which represents a greater than fourfold increase in comparison to 2018 (1). In the realm of musculoskeletal and soft tissue tumor imaging, there has been increasing enthusiasm for a broad range of AI applications ranging from prognostication and risk stratification to lesion classification and treatment response (Varghese et al., 2–4).

This Research Topic, titled “*Advances in Artificial Intelligence and Machine Learning Applications for the Imaging of Bone and Soft Tissue Tumors*,” encompasses a selection of manuscripts that primarily focus on recent developments in AI applications targeting the imaging of these neoplasms while also exploring topics related to quantitative image analysis and interpretation. The following representative manuscripts provide insights into current advancements and methodologies within this evolving field:

Jin et al. describe a machine learning algorithm based on Gray Level Co-occurrence Matrix (GLCM) scores to detect pelvic bone metastases in patients with colorectal cancer using a retrospective cohort of 614 patients who underwent MRI of the pelvis over a 7-year period. Diffusion-weighted images were segmented, from which 48 GLCM features were extracted for further analysis. A generalized linear regression model and four machine learning classification models were then constructed from this dataset. Random forest achieved the highest performance, with AUCs of 0.926 and 0.919 in the training and internal validation sets, respectively. Their results suggest a promising role for radiomics-based machine learning models in detecting pelvic bone metastases. By integrating these advanced modeling techniques into clinical workflows, healthcare providers may enhance their ability to identify osseous metastatic disease more reliably, thereby potentially improving prognostic assessments and guiding treatment interventions tailored to individual patient needs.

Rich et al. publish a systematic review and meta-analysis of deep learning image segmentation approaches for the evaluation of primary and secondary malignant bone tumors. Accurate delineation of osseous lesions on imaging is crucial for performing quantitative analyses, yet is often the rate-limiting step in AI and machine learning studies when performed manually. Additionally, precise delineation of the extent of bony involvement of disease may allow for more holistic clinical assessments and prognostication in situations of widespread metastatic disease. In their article, Rich et al. conducted a comprehensive literature search of studies investigating automated deep-learning segmentation methods for malignant bony lesions, identifying 41 studies published between 2010 and 2023 suitable for inclusion in the final analysis. Overall, they found that most studies applied a U-Net convolutional neural network architecture, most often trained on either MRI or CT images. Models trained on PET/MRI and PET/CT had lower median dice similarity coefficients when compared to models trained on MRI and CT, possibly due to increased image noise and degradation. Furthermore, models trained on 2D data appeared to have slightly higher median dice similarity coefficients, though the difference was not statistically significant. Future optimizations and validations could include training of models on larger, multi-institutional datasets to allow for improved generalizability.

Debs and Fayad and Sabeghi et al. both describe emerging applications of AI and machine learning in musculoskeletal imaging. Debs and Fayad review use cases ranging from image protocoling, examination scheduling, and hanging protocol optimization to results reporting, lesion detection, and lesion classification. They also detail approaches for determining tumor of origin and assessing for treatment response of metastatic spinal lesions. Sabeghi et al. comment on methods for stratifying neoplastic lesions with respect to malignant potential, histologic grade, and response to treatment. They further discuss challenges with assembling sufficiently large, diverse, and heterogeneous datasets to train robust, generalizable models. As models become increasingly more complex and advanced, federated learning may offer an elegant solution for leveraging multicentric training sets without the need for centralized aggregation of potentially sensitive and protected patient data (5), though technical limitations and complexities associated with managing heterogeneous systems and data pose major hurdles.

Gadermayr et al. investigate computer vision and deep learning approaches for automated muscle segmentation of MR images. The authors employ an unpaired image-to-image translation approach to leverage “easy” data in order to improve segmentation performance of “hard” data. Using a novel domain specific loss function alongside four segmentation schemes, namely Gaussian mixture, graph-cut, shape-priority graph-cut, and convolutional neural network approaches. Their results suggest performance benefits for both unsupervised and supervised methods, with the potential to reduce the amount of necessary training data in related studies.

Woznicki et al. developed an open-source framework termed “AutoRadiomics”, which seeks to aggregate all common steps of typical radiomics workflows into a single standardized software package. Their framework provides embedded tools for image segmentation and pre-processing alongside standardized radiomics libraries and machine learning models, and provides multiple optimizations for hyperparametric tuning, data splitting, and oversampling. AutoRadiomics seeks to lower the barrier to entry for radiomics and machine learning studies through a modular, user-friendly interface and intuitive design, promising accessibility without the need for a robust coding background. Consistent application of publicly accessible frameworks such as AutoRadiomics in future radiomics studies can serve to enhance transparency by improving workflow standardization and reproducibility.

Finally, Varghese et al. discuss applications of spatial assessments of texture analysis in oncologic imaging. Spatial assessments are particularly effective in capturing areas of intratumoral heterogeneity as they have the ability to quantify subtle voxel-to-voxel variations in the underlying grayscale intensities. This granular approach allows for a more nuanced understanding of tumor characteristics, which is critical for accurate diagnosis and treatment planning. These spatial assessments can be further classified as neighborhood-based methods, which quantify differences in grayscale intensities relative to neighboring voxels, and spatial filters, which are image processing methods that enhance edges and/or textures at regions of rapid grayscale change. Additionally, the authors also underscore the significance of utilizing optimal methodologies and best practices when conducting statistical analyses in high-dimensional radiomics studies. They offer guidance on accurate reporting of machine learning performance outcomes to ensure that findings are both reliable and reproducible.

In conclusion, this Research Topic highlights many innovative developments in AI and machine learning applications for the imaging of bone and soft tissue neoplasms. While radiomics-based studies have for some time shown promise in serving as novel quantitative imaging biomarkers, deep learning approaches have, in more recent years, also gained traction as powerful decision support tools which integrate diverse hierarchical and multimodal inputs. As imaging-based AI algorithms continue to evolve in complexity and rigor, there exists immense potential for creating an armamentarium of tools aimed at augmenting the work of clinical radiologists and enhancing patient care.
